# Research on the resistance of isoviolanthin to hydrogen peroxide-triggered injury of skin keratinocytes based on Transcriptome sequencing and molecular docking

**DOI:** 10.1097/MD.0000000000036119

**Published:** 2023-11-24

**Authors:** Jie Wang, Hao Yin, Wei Zhu, Qingyi He, Haitang Zhang, Lu Sun, Yunxiao Qiao, Yanwei Xiang

**Affiliations:** a School of Rehabilitation Science, Shanghai University of Traditional Chinese Medicine, Shanghai, China; b Institute of Vascular Disease, Shanghai TCM-Integrated Hospital, Shanghai University of Traditional Chinese Medicine, Shanghai, China; c Department of Dermatology, Yueyang Hospital of Integrated Traditional Chinese and Western Medicine, Shanghai University of Traditional Chinese Medicine, Shanghai, China; d Engineering Research Center of Traditional Chinese Medicine Intelligent Rehabilitation, Ministry of Education, Shanghai, China.

**Keywords:** cell apoptosis, isoviolanthin, p53 signaling pathway, RNA sequencing

## Abstract

Apoptosis of skin keratinocytes is closely associated with skin problems in humans and natural flavonoids have shown excellent biological activity. Hence, the study of flavonoids against human keratinocyte apoptosis has aroused the interest of numerous researchers. In this study, methyl thiazolyl tetrazolium (MTT) assay and Western blots were used to investigate the skin-protective effect of isoviolanthin, a di-C-glycoside derived from *Dendrobium officinale*, on hydrogen peroxide (H_2_O_2_)-triggered apoptosis of skin keratinocytes. Transcriptome sequencing (RNA-Seq) was used to detect the altered expression genes between the model and treatment group and qRT-PCR was used to verify the accuracy of transcriptome sequencing results. Finally, molecular docking was used to observe the binding ability of isoviolanthin to the selected differential genes screened by transcriptome sequencing. Our results found isoviolanthin could probably increase skin keratinocyte viability, by resisting against apoptosis of skin keratinocytes through downregulating the level of p53 for the first time. By comparing transcriptome differences between the model and drug administration groups, a total of 2953 differential expression genes (DEGs) were identified. Enrichment analysis showed that isoviolanthin may regulate these pathways, such as DNA replication, Mismatch repair, RNA polymerase, Fanconi anemia pathway, Cell cycle, p53 signaling pathway. Last, our results found isoviolanthin has a strong affinity for binding to KDM6B, CHAC2, ESCO2, and IPO4, which may be the potential target for treating skin injuries induced by reactive oxide species. The current study confirms isoviolanthin potential as a skin protectant. The findings may serve as a starting point for further research into the mechanism of isoviolanthin protection against skin damage caused by reactive oxide species (e.g., hydrogen peroxide)

## 1. Introduction

The skin is mainly composed of epidermis, dermis, and subcutaneous tissue. It defends the body from environmental chemicals, physical invasion, and pathogen damage, as well as regulates body temperature to prevent the loss of uncontrollable water and solutes.^[1]^ Last but not least, it is essential for the perception of vitamin D and vitamin D synthesis.^[2]^ Coincidentally, a new study finds the level of cystatin A in human skin epidermal keratinocytes could meditate age-related bone loss.^[3]^ As the body largest barrier organ, the skin epidermis is constantly exposed to various pathogenic factors, such as daily solar radiation (especially UVA, UVB, and visible light), air pollution, tobacco smoking, nutrition,^[4,5]^ which account for the skin oxidative stress, DNA damage and skin aging.^[6]^ The outermost layer of the skin is mainly formed by keratinocytes, which account for approximately 95% of all cells in the epidermis.^[7,8]^ In epidermal keratinocytes, filaggrins and claudins play a vital role in maintaining the skin barrier.^[9]^ Additionally, keratinocyte aquaporins with effects on hydration, permeability barrier repair, and wound healing also play a pivotal role in the epidermis.^[10]^ In light of this, skin damage and aging are gaining increasing concern in modern society. It is urged that the development and application of skin protective agents should be accelerated.

*Dendrobium officinale*, a famous and precious traditional Chinese medicine originally recorded in *Sheng Nong herbal classic*, has been used as a medicinal and food homologous for thousands of years in China and Southeast Asia. Previous research showed that it has antioxidant, anti-inflammatory, and skin protection effects.^[11,12]^ Many studies focused on the chemical analysis and pharmacological effects of polysaccharides and stilbenes of *Dendrobium officinale*,^[11,13–15]^ with little attention paid to its flavonoids. However, its stems, leaves, and flowers were rich in flavonoids, most of which were in the form of C-glycosides.^[16]^ Among the flavonoids, the isoviolanthin is considered as the specific component of *Dendrobium officinale* to be distinguished from other dendrobium because it is only shared by *Dendrobium officinale* from different regions and different parts.^[17,18]^ Furthermore, it reported that flavone C-glycoside from *Dendrobium officinale* could scavenge DPPH free radicals’ activity.^[19]^

It reported increasingly that flavonoids from plants have good antioxidant capacity,^[20]^ anti-aging effects^[21,22]^, and skin protection.^[2,23]^ Similarly, the flavonoid isoviolanthin found in the plant *Dendrobium officinale* exhibits antioxidant properties. Therefore, this study investigated for the first time the skin protective effects of isoviolanthin in *Dendrobium officinale* based on RNA-Seq and molecular docking method, which may lay a foundation for further research into the application of isoviolanthin as a skin protectant and the relevant mechanisms.

## 2. Materials and methods

### 2.1. Isoviolanthin and hydrogen peroxide

**Figure 1.**
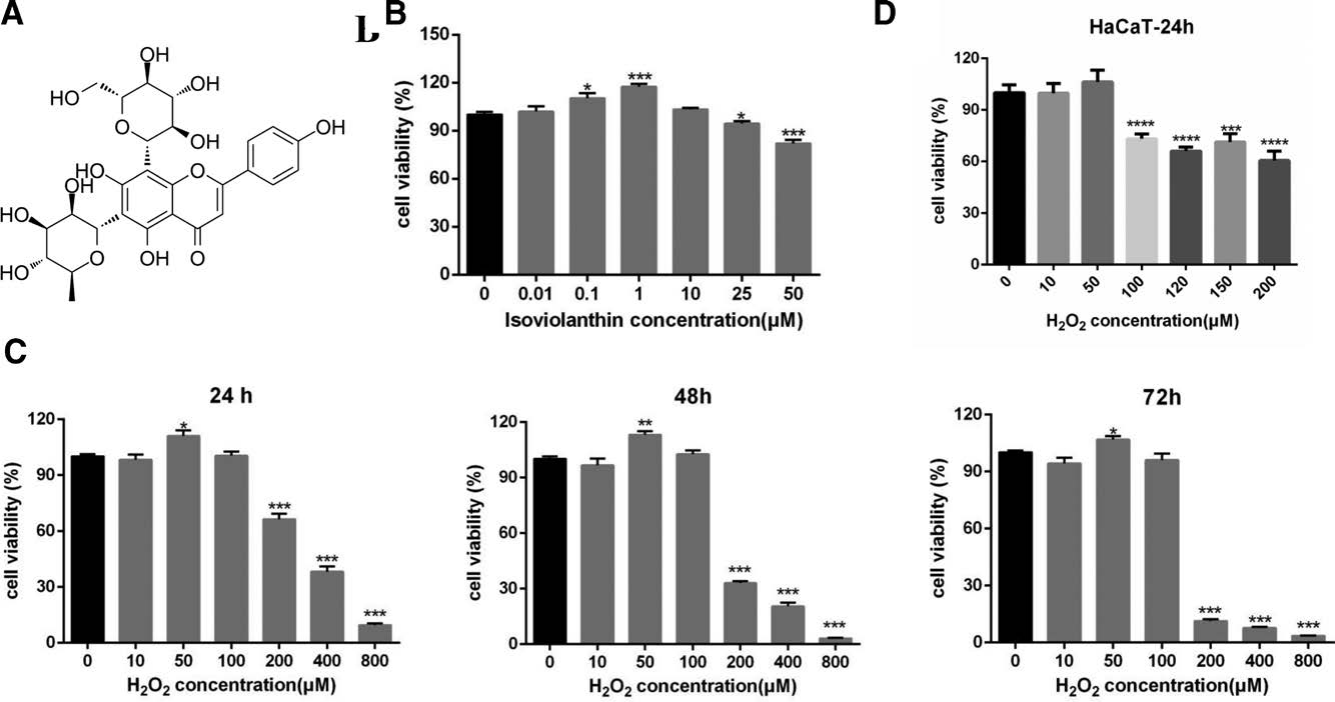
Toxicity of isoviolanthin on the HaCaT cell and the construction of skin keratinocyte injury model. (A) The chemical structure of isoviolanthin used in this study; (B) The toxic effect of various concentration of isoviolanthin on HaCaT was detected by MMT assay. (C) Effect of different concentrations of H_2_O_2_ on the cell viability of HaCaT cells when co-incubated with HaCaT cells for 24h, 48h, and 72h, respectively. (D) Establishment of skin keratinocyte injury model by 120 μM H_2_O_2_. Results are presented as the mean ± SD of 4 independent experiments.

Isoviolanthin (Fig. 1A), was purchased from Chengdu Biopurify Phytochemicals Ltd (Sichuan, China), configured with DMSO to 200 mM, stored at −20°C, protected from light, and set aside. Hydrogen peroxide solution (500 mL, 3%) was purchased from Sinopharm Chemical Reagent Co., Ltd. and stored at room temperature.

### 2.2. The immortalized human keratinocyte (HaCaT) culture

HaCaT keratinocytes were purchased from the National Collection of Authenticated Cell Cultures (Shanghai, China) and kept in a high-glucose Dulbecco modified Eagle medium (DMEM, BBI) containing fetal bovine serum (10%, V/V) and 0.1% penicillin–streptomycin, then kept at 37°C in a humidified 5% CO_2_ incubator. Cell experiments begin when the cell confluence is about 80%.

### 2.3. Cell viability assay

HaCaT cell viability was determined by methyl thiazolyl tetrazolium (MTT) assay. Firstly, the MTT solution containing DMEM (DMEM: MTT = 9:1) was prepared, and 100 μL of MTT solution was added into each group of a 96-well plate, and then incubated in a cell incubator (37°C with 5% CO_2_) for 3h. Subsequently, the supernatant of each well was discarded and 100 μL of DMSO was added to dissolve the formazan crystals, Lastly, the absorbance values of the cells were measured at 492 nm with a microplate reader (SAF-680T, Bajiu Industry Co., LTD).

### 2.4. Cytotoxicity assay of isoviolanthin

The HaCaT keratinocytes (100 μL) were seeded onto 96-well plates containing 3 × 10^3^ cells per well. The following day, isoviolanthin was dosed at 7 concentrations (0, 0.01, 0.1, 1, 10, 25, 50 μM) and each concentration was performed 4 times. On the fifth day, the supernatant was discarded, and 100 μL of MTT solution was added to each well. Cell viability was then determined using the MTT method.

### 2.5. Skin keratinocyte injury model induced by hydrogen peroxide

The HaCaT keratinocytes were seeded simultaneously onto 3 96-well plates (3 × 10^3^ cells/well) overnight, and the cells were treated with different concentrations of hydrogen peroxide on the following day, the same for the 3 96-well plates. Finally, the viability of the cells was determined using the MTT method on the third day (24 hours), fourth day (48 hours) and fifth day (72 hours), respectively.

### 2.6. Effects of isoviolanthin on H_2_O_2_-induced skin keratinocyte injury

The HaCaT keratinocytes were seeded onto 96-well plates containing 3 × 10^3^ cells per well. The supernatant was discarded the following day and 120 μM hydrogen peroxide (100 μL) was added to each well. On the third day, cells were divided into 5 groups, and each group was treated with different concentrations (0, 0.01, 0.1, 1, 10 μM) of isoviolanthin. Finally, the viability of the cells was determined using the MTT method on the fifth day.

### 2.7. Protein extraction and Western blotting

According to the results of experiment *2.6*, a total of 4 experiment group (n = 4) were divided to observe the expression of p53 in HaCaT cells using Western blots, as follows: control group; 100 μM H_2_O_2_ group; 200 μM H_2_O_2_ group; 100 μM H_2_O_2_ and 1 μM isoviolanthin group. Cold RIPA buffer containing a protease inhibitor cocktail (Beyotime Biotechnology, China) was used to lyse the treated HaCaT keratinocytes. Furthermore, the protein content of cell homogenates was determined using a BCA Protein Assay Kit (Thermo Fisher Scientific Inc.) following the manufacturer protocol.

Sodium dodecyl sulfate − polyacrylamide gel electrophoresis (SDS-PAGE) was used to separate approximately 20 μg of protein, which was then transferred onto an immobile polyvinylidene fluoride (PVDF) membrane (Millipore). The PVDF membranes were blocked for 2 hours at room temperature with 5% skim milk. The membranes were then incubated with specific primary antibodies, including anti-GAPDH (1:10000) and anti-p53 (1:2000). For 10 minutes, the PVDF membranes were washed 3 times with phosphate-buffered saline Tween-20 (TBST). It was then incubated for 2 hours at room temperature with the secondary antibody. An ECL Western Blot Detection Kit (Beyotime Biotechnology, China) was used to visualize protein bands. Using ImageJ 1.5.1 analysis software, each plot was imaged, quantified, and expressed as the relative density (%).

### 2.8. Transcriptome sequencing (RNA-Seq) of Isoviolanthin treated injured HaCaT cells induced by hydrogen peroxide

The protective effect of isoviolanthin on injured skin keratinocytes has been confirmed by MTT and Western blotting experiments, so we extracted total RNA from keratinocytes treated by isoviolanthin for 72h using RNeasy mini kit (QIAGEN, Germany). Following purification, the mRNA is fragmented into small pieces using divalent cations under 94°C for 8 minutes. The cleaved RNA fragments are copied into first-strand cDNA using reverse transcriptase and random primers. This is followed by second-strand cDNA synthesis using DNA Polymerase I and RNase H. These cDNA fragments then go through an end repair process, the addition of a single “A” base, and then ligation of the adapters. The products are then purified and enriched with PCR to create the final cDNA library. Purified libraries were quantified by Qubit® 2.0 Fluorometer (Life Technologies, Germany) and validated by Agilent 2100 bioanalyzer (Agilent Technologies) to confirm the insert size and calculate the mole concentration. Paired-end transcriptome sequencing was carried out at Sinotech Genomics Co., Ltd. Clusters were generated by cBot using a diluted library of 10 pM and then sequenced on the Illumina NovaSeq 6000 (Illumina, USA). HISAT2 was used to map paired-end sequence files to the reference genome (GRh38-1.2.0).^[24]^ Differential expression of mRNAs was analyzed using the R package (version 3.4.3) edgeR, where differentially expressed RNAs with |log_2_ (FC) | values >1 and *adjusted p* values <.05, were considered as significantly regulated genes. Then these genes were subjected to volcano map analysis, GO, and KEGG analysis. The raw transcriptome data of this study were stored in the National Omics Data Encyclopedia database repository “OEP003865.”

### 2.9. GO and KEGG pathway enrichment analysis

To classify the biological functions and signaling pathways that the differential expression genes (DEGs) are involved in, we annotated DEGs based on GO and KEGG databases (https://www.genome.jp/). When the species under study had a relevant GO annotation database, GO analysis was performed directly using the database. If not, Blast2GO (https://www.blast2go.com/) was used to obtain the corresponding GO entry for each gene. The number of DEGs in the secondary GO entries (such as cellular component, biological process, and molecular function) was calculated. Compared with genome background,^[25]^ GO enrichment analysis can determine which biological functions are closely associated with DEGs. Meanwhile, different genes collaborate in vivo to elicit a variety of biological functions, such as cell division, protein synthesis, and cell signaling pathways, which can be determined using KEGG pathway significance enrichment.^[26]^ The functional annotation of target genes obtained through KEGG enrichment was analyzed in this study using The Database for Annotation, Visualization, and Integrated Discovery (DAVID) Bioinformatics Resources 6.8 (https://david.ncifcrf.gov). The gene symbol was entered into the DAVID online tool to get functional items.

### 2.10. RNA extraction and RT-PCR

HaCaT cells at the exponential growth phase were selected and seeded evenly into 6-well plates with 2 × 10^5^ cells/well. Cultured overnight, the cells were divided into the hydrogen peroxide treatment group and hydrogen peroxide (100 μM) + isoviolanthin (1 μM) treatment group (n = 3). RNA was extracted after exposure to H_2_O_2_ within 24 hours and isoviolanthin within 72 hours. According to the guidance manual, the RNAprep Pure cell Kit (Tiangen Biotech Co., Ltd., China) was used for total RNA extraction. The concentration and purity of total RNA were determined by Nano-100 Micro-Spectrophotometer (Hangzhou Allsheng Instruments Co., Ltd.), and total RNA with a purity of 1.8 to 2.1 was obtained for further application. The cDNA was prepared by the First Strand cDNA Synthesis Kit (TOYOBO CO., Tokyo, LTD, Japan). The real-time quantitative PCR primers listed in the Table were synthesized by Sangon Biotech (Shanghai) Co., Ltd. The RT-PCR reaction system was 20 μL, including SYBP Green Mix 10 μL, forward primer 1 μL, reverse primer 1 μL and cDNA 1 μL, and ultrapure water 7 μL. Real-time fluorescence quantitative PCR (Heal Force Bio-Meditech Holdings Limited, Shanghai) was used to make PCR reaction with the normal condition (pre-denaturation at 95°C for 60 seconds, then denaturation at 95°C for 15 seconds, annealing at 60°C for 60 seconds, and a total of 40 cycles). Gene expression was calculated by applying the 2^-∆∆Ct^ method using GAPDH as the reference gene.

### 2.11. Molecular docking

PubChem database (https://pubchem.ncbi.nlm.nih.gov/) was used to obtain the SDF format file of isoviolanthin, and Chem3D 15.1 software was used to optimize the energy of isoviolanthin, which is subsequently saved as a “mol2” format file. Receptor structure in PDB format was downloaded from the RCSB PDB database (https://www.rcsb.org/), that is Lysine-specific demethylase 6B (KDM6B) (PDB ID:5OY3), Glutathione-specific gamma-glutamylcyclotransferase 2(CHAC2) (PDB ID:6K95), N-acetyltransferase ESCO2 (ESCO2) (PDB:6SP0), Importin-4 (IPO4) (PDB ID:5XAH), then all ligands and solvents of the molecule were removed by PyMOL 2.5 software. Finally, AutoDock Tools-1.5.6 software was used for molecular docking simulation when the PDBQT format file of ligand and receptor were ready. The target protein was taken as the center of the grid, while the parameters of the center coordinates (center X/Y/Z) were predicated by DeepSite database (https://www.playmolecule.com/deepsite/), respectively, KDM6B (86.8, −8.9, 22.7), CHAC2 (68.56, −10.66, 55.40), ESCO2 (19.1, 45.8, 68.1), and IPO4 (−18.8, 75.1, 43.2). The box size (size X/Y/Z) was adjusted to ensure that the protein macromolecules were completely covered by the docking box before molecular docking was conducted. Then PyMOL 2.5 and LigPlot + (version 2.2.8) were used for molecular docking result processing and visual analysis.^[27]^ If the binding energy is less than −5 kcal/mol,^[28,29]^ it is considered that the docking result between ligand and receptor protein is stable and acceptable.

### 2.12. Statistical analyses

All data, presented as mean ± standard deviation (SD), were entered into GraphPad Prism software (San Diego) for analysis. Student *t* test was used to analyze the difference in cell viability between the administered and control groups. Multiple group comparisons were performed statistically using a one-way analysis of variance(ANOVA)followed by Tukey post hoc test. When the significant difference between the administered group and the control group was <0.05, it was considered to be a significant difference. *, **, *** and **** represent *P<*.05, *P<*.01, *P<*.001 and *P<*.0001, respectively.

## 3. Results

### 3.1. Cytotoxicity assay of isoviolanthin on HaCaTs and establishment of skin keratinocyte injury model

To determine whether isoviolanthin has toxicity on HaCaT cells, we used the MTT assay to examine the viability effects of different concentrations (0, 0.01 μM, 0.1 μM, 1 μM, 10 μM, 25 μM, 50 μM) of isoviolanthin on HaCaT cells. As shown in Figure 1B, surprisingly, isoviolanthin at 0.1 μM and 1 μM could significantly promote HaCaT proliferation, whereas the pro-proliferative effects of 0.01 μM and 10 μM isoviolanthin on HaCaT were not significantly different from the control group. The 0.1 μM and 1 μM isoviolanthin groups resulted in 110.2% and 117.4% cell viability, respectively. When compared to the control group, isoviolanthin showed a dose-dependent promotion of HaCaT viability at 0.01 to 1 μM. When isoviolanthin concentrations were up to 25 μM and 50 μM, it inhibited cell viability, reducing cell viability to 94.41% and 82.02%, respectively. According to the findings, isoviolanthin may promote the growth of skin keratinocytes. In the following experiment, we chose isoviolanthin with a concentration of 0.01 μM, 0.1 μM, 1 μM, and 10 μM to see if it has a protective effect on damaged skin keratinocytes.

To confirm the beneficial effect of isoviolanthin on injured skin keratinocytes, we treated HaCaTs with H_2_O_2_ at concentrations ranging from 0 to 800 μM, and then cultured HaCaTs for 24 hours, 48 hours, and 72 hours to tentatively construct an H_2_O_2_-induced skin keratinocyte injury model and detect their cell viability using the MTT assay. As shown in Figure 1C, the cell viability decreased dramatically when the H_2_O_2_ concentration was greater than or equal to 200 μM at 24 hours, 48 hours, and 72 hours. Therefore, the appropriate inhibitory concentration of H_2_O_2_ should lie between 100 and 200 μM. Eventually, we selected 120 μM H_2_O_2_ co-cultured with keratinocytes for 24 hours to establish an H_2_O_2_-triggered skin keratinocyte injury model (Fig. 1D).

### 3.2. Isoviolanthin favors HaCaT cell viability in H_2_O_2_-triggered injured skin keratinocytes

**Figure 2.**
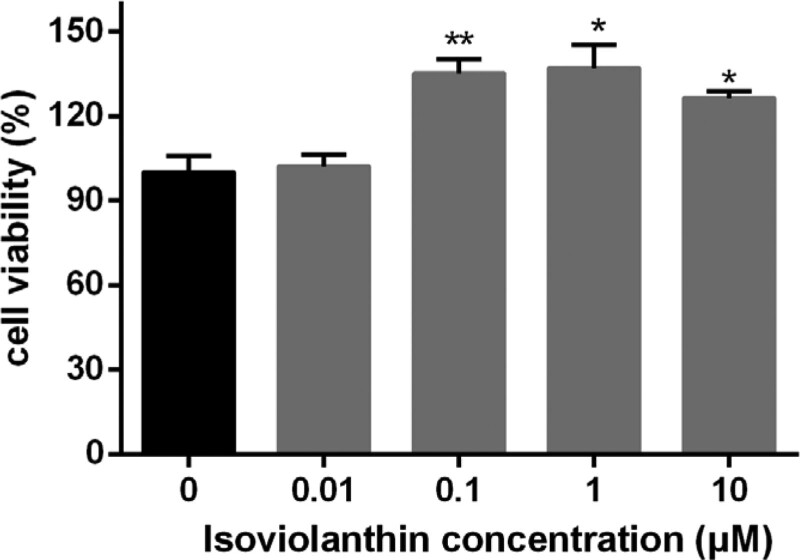
Protective effect of isoviolanthin on HaCaT keratinocyte injury induced by H_2_O_2_.

In the experimental group, all groups were pretreated with 120 μM hydrogen peroxide, then cultured with 5 different concentrations of isoviolanthin to observe the protective effect on the injured skin keratinocytes induced by hydrogen peroxide. Figure 2 shows that 0.1, 1, and 10 μM isoviolanthin could significantly promote the cell viability induced by hydrogen peroxide, and the effect was the best at concentrations of 1 μM. This illustrates that isoviolanthin could resist H_2_O_2_-triggered injured skin keratinocytes.

### 3.3. Isoviolanthin down-regulates the expression of P53 in H_2_O_2_-induced injured skin keratinocytes

**Figure 3.**
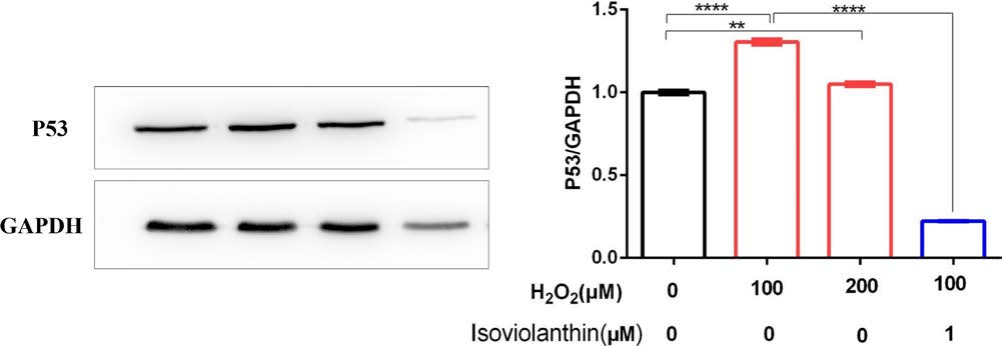
Effects of isoviolanthin on the expression of total p53 protein in normal HaCaT cells and injured HaCaT cells. On the right, the protein expression of p53 in HaCaT cells treated with H2O2 with or without isoviolanthin was detected by western blotting. On the right, the protein band intensity of each blot was quantitated and normalized based on that of the GAPDH blot.

It is well-known that p53 protein plays an eminently important role in the induction of cell death.^[30,31]^ Under non-stressed conditions, p53 levels in normal cells are maintained at very low levels, because p53 is targeted for proteasomal degradation by the E3 ubiquitin ligase MDM2; When cells are under stress or damage conditions, the levels of p53 protein rise substantially.^[32]^ Therefore, we investigated whether isoviolanthin inhibits the expression of the p53 protein induced by H_2_O_2_. As shown in Figure 3, 100 and 200 μM H_2_O_2_ promoted the expression of p53 protein remarkably. However, as shown in the same figure, 1 μM isoviolanthin reversed the effect of H_2_O_2_ on the p53 protein conspicuously. This indicates that isoviolanthin could inhibit the expression of p53 protein to exert an anti-apoptosis effect in HaCaT keratinocytes, thereby protecting human keratinocyte injury.

### 3.4. Isoviolanthin altered the transcriptional gene expression in injured HaCaT keratinocytes

**Figure 4.**
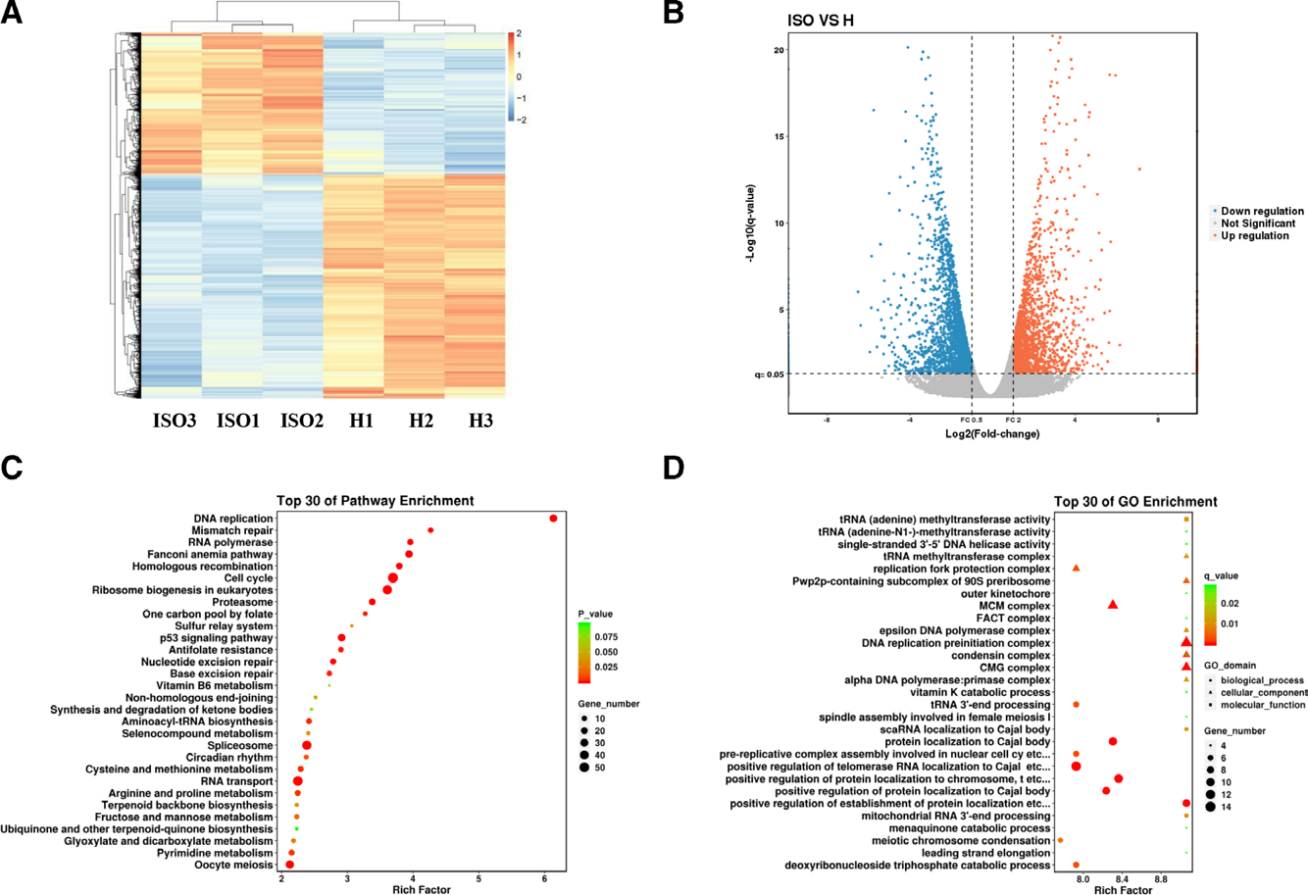
Analysis of changed genes, gene ontology, and biological pathways involved in isoviolanthin exerted inhibition on injured HaCaT keratinocytes. (A) Heatmap of differentially expressed genes between the “H” group and “ISO” group. The color orange signifies abundantly expressed genes, while the color light blue reflects poorly expressed genes. (B) Volcano plot of differentially expressed genes between the “H” group and “ISO” group. The orange points within the boundary were upregulated genes, the light blue points were downregulated genes, and the gray represents non-significant differential genes. (C) Biological pathway analysis of isoviolanthin to injured HaCaT keratinocytes induced by H2O2. (D) GO enrichment of potential targets of isoviolanthin for injured skin keratinocytes. HaCaT = The Immortalized Human Keratinocyte.

As aforementioned in *3.2* and *3.3*, we tested the biological effects of the isoviolanthin on injured HaCaT keratinocytes induced by H_2_O_2_. Furthermore, to explore the potential molecular mechanisms of isoviolanthin protection of injured keratinocytes, transcriptomic profiling was used to compare the differentially expressed gene profiles of the isoviolanthin-treated injured HaCaT keratinocytes group (ISO group) and the H_2_O_2_-treated cells (H group). Correlation analysis of sequencing samples (Supplementary Figure S1, http://links.lww.com/MD/K763) revealed that duplicate samples were highly similar and reproducible. Significant differences between the “H group” and “ISO group” at the transcriptome level can be seen from the principal component analysis diagram (Supplementary Figure S2, http://links.lww.com/MD/K764). Based on transcriptome sequencing and the above-mentioned DEGs screening criteria, isoviolanthin treatment affects more than 2953 DEGs in HaCaT cells, including 1148 upregulated genes and 1805 downregulated genes (Supplementary, Table S1, http://links.lww.com/MD/K761 and S2, http://links.lww.com/MD/K762), and a heat map (Fig. 4A) and a volcano map (Fig. 4B) of all the DEGs was generated.

Immediately afterward, the DAVID database was used to analyze the DEGs to get more information about the relevant biological processes. Our findings shed new light on multiple KEGG pathways (Fig. 4C) that isoviolanthin regulated, such as DNA replication, Mismatch repair, RNA polymerase, Fanconi anemia pathway, cell cycle, and p53 signaling pathway, which were immensely related to cellular injury. Among them, the p53 signaling pathway was partially verified by the results of experiment *3.3*. Functional analysis (Fig. 4D) of all the DEGs affected by isoviolanthin showed that positive regulation of establishment of protein localization to telomere, positive regulation of protein localization to chromosome, telomeric region, protein localization to Cajal body were the main biological processes in the regulation of injured skin keratinocytes. Isoviolanthin treatment also affected several cellular components important for skin keratinocyte proliferation, including the MCM complex, DNA replication preinitiation complex, and CMG complex. According to molecular function analysis, the tRNA (adenine) methyltransferase activity, tRNA (adenine-N1-)-methyltransferase activity, and single-stranded 3’-5’ DNA helicase activity were all notably changed when treated with isoviolanthin.

**Figure 5.**
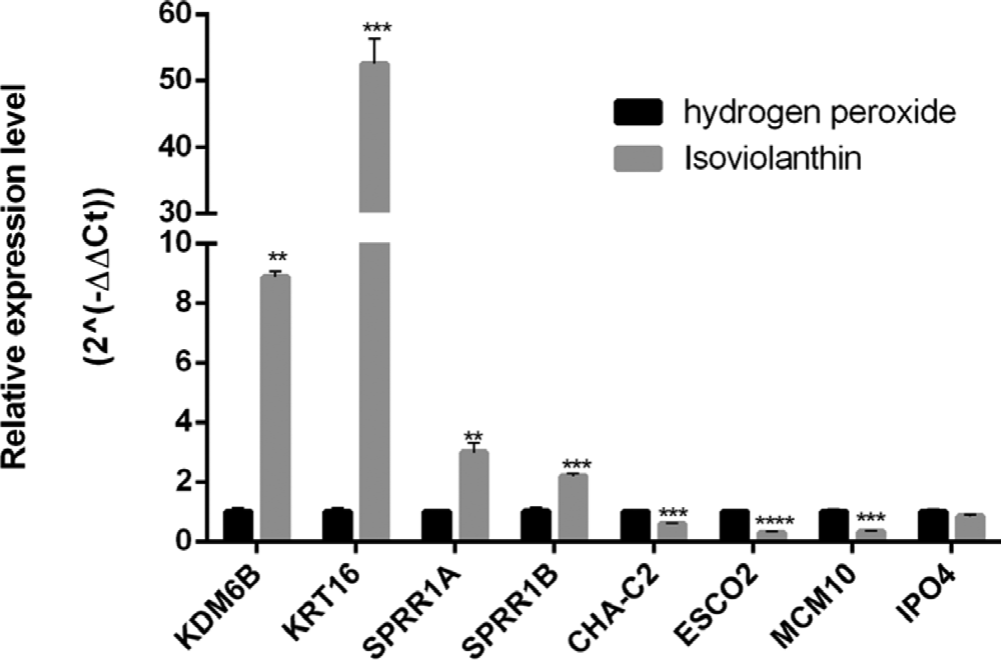
qRT-PCR verification of 8 mRNA from H and ISO group.

Among the 2952 differential genes, we filtered the top 30 mRNA of up-regulated and down-regulated genes according to |log2 (FC) | values and then retrieved the literature on whether these mRNAs were related to cell proliferation and apoptosis. Finally, 4 genes were identified in each of the up-regulated and down-regulated genes for qRT-PCR validation and the gene prime sequences are shown in Table 1. As shown in the Figure 5, the expression levels of *KDM6B, KRT16, SPRR1A,* and *SPRR1B* were significantly up-regulated and the levels of *CHAC2, ESCO2, IPO4,* and *MCM10* were significantly down-regulated in the isoviolanthin-treated group compared with the model group. Meanwhile, the results were remarkably consistent with the transcriptome sequencing results (Table S1 and Table S2). Among them, the expression trends of *KRT16, SPRR1A, SPRR1B,* and *MCM10* were also consistent with previous literature.^[33–36]^

**Table 1:** Real time RT-PCR primers used for quantification of mRNA.

Name of mRNA		Sequences (5’-3’) of used primers
KDM6B	Forward	CACCCCAGCAAACCATATTATGC
	Reverse	CAC ACA GCC ATG CAG GGA TT
KRT16	Forward	GACCGGCGGAGATGTGAAC
	Reverse	CTGCTCGTACTGGTCACGC
SPRR1A	Forward	GGTGAAACAACCTTGCCAG
	Reverse	TTCTGCTTGGTCTTCTGCT
SPRR1B	Forward	CTCTTCACACCAGGACCAG
	Reverse	CATGGTTCCTGAGGTGGAG
CHAC2	Forward	GGTTTTTGGTTACGGGTCCCT
	Reverse	GCAACACCCCATACACATCCC
ESCO2	Forward	CACTGGGACGCACCCAAAA
	Reverse	CACTTGCCTTGTCGCAAAAG
MCM10	Forward	TGTCCCTGCGCTACCAAGA
	Reverse	GATGAGCTTTTGGGATCTGGAG
IPO4	Forward	GCTCCAGATCGTTCTTCGGG
	Reverse	CCGTCAGGATCAGGGACTTG

CHAC2 = Glutathione-specific gamma-glutamylcyclotransferase 2, ESCO2 = N-acetyltransferase ESCO2, IPO4 = Importin-4, KDM6B = Lysine-specific demethylase 6B.

### 3.5. Isoviolanthin binds to KDM6B, CHAC, ESCO2 and IPO4 Protein

Of the 8 genes in Table 1, only 4 have the corresponding protein crystal structure-based experiment. So we investigated the molecular docking between isoviolanthin and these 4 genes (*KDM6B, CHAC2, ESCO2, IPO4*).

**Figure 6.**
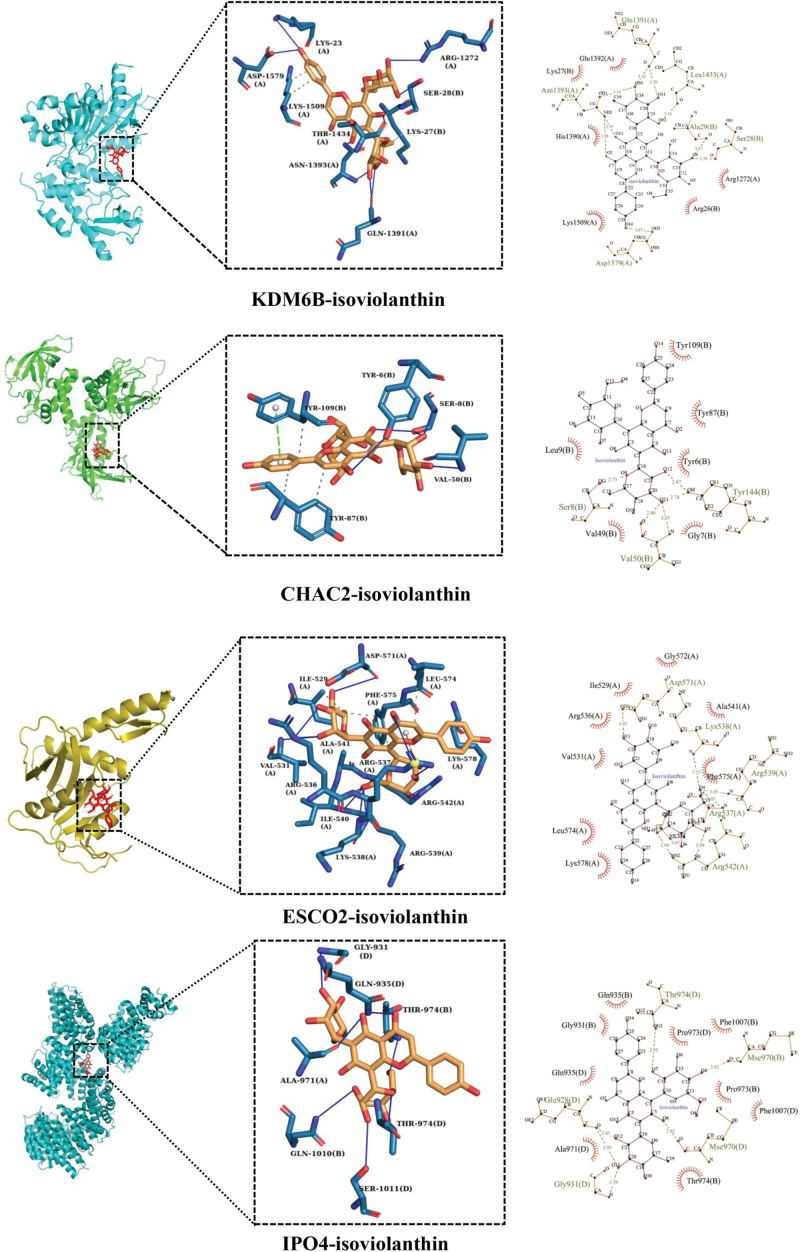
Molecular docking of isoviolanthin with KDM6B, CHAC2, ESCO2 and IPO4. The first 2 diagrams of each molecular docking were visualized by PyMOL, and the last diagram was drawn by LigPlot. CHAC2 = Glutathione-specific gamma-glutamylcyclotransferase 2, ESCO2 = N-acetyltransferase ESCO2, IPO4 = Importin-4, KDM6B = Lysine-specific demethylase 6B.

As seen in Figure 6, Isoviolanthin formed hydrogen bonds with KDM6B residues Gln1391, Leu1434 (A), Asn1393 (A), Asp1579 (A), Arg1272 (A), and Ser28, which aid in the stability of the structure. In addition, isoviolanthin formed hydrogen bonds with CHAC2 residues Ser8 (B) and Val50 (B), which contribute to the stability of isoviolanthin and CHAC2 binding. Isoviolanthin formed hydrogen bonds with ESCO2 residues Asp571 (A), Arg539 (A), Arg537 (A) and Arg542 (A). Isoviolanthin formed hydrogen bonds with Gly931 (D), Thr974 (D), Gln1010 (B), and Ser1011 (D) in IPO4. It can be seen that isoviolanthin has a strong affinity for binding to KDM6B, CHAC2, ESCO2, and IPO4 because their binding energy shown in Table 2 is less than -5 kcal/mol.

**Table 2:** The binding energy of isoviolanthin with 4 gene targets.

Compound	KDM6B (Kcal/mol)	CHAC2 (Kcal/mol)	ESCO2 (Kcal/mol)	IPO4 (Kcal/mol)
Isoviolanthin	−8.90	−8.00	−9.10	−9.20

CHAC2 = Glutathione-specific gamma-glutamylcyclotransferase 2, ESCO2 = N-acetyltransferase ESCO2, IPO4 = Importin-4, KDM6B = Lysine-specific demethylase 6B.

## 4. Discussion

The skin cells most sensitive to environmental factors are epidermal keratinocytes, which could be induced to produce hydrogen peroxide by solar ultraviolet radiation.^[5]^ Subsequently, considerable hydrogen peroxide can upregulate cellular oxidative stress, increase p53 expression, and promote cell apoptosis and carcinogenic effects.^[37]^ Reactive oxygen species (ROS), such as hydrogen peroxide derived from mitochondrial respiration, have been mainly regarded as a major source of cellular damage to DNA and skin macromolecules.^[38]^ According to the MTT assay, the H_2_O_2_-induced skin keratinocyte injury model is successfully established, which is similar to the previous study.^[39]^ This study is the first to find that isoviolanthin could significantly alleviate the skin keratinocyte apoptosis induced by H_2_O_2_ and remarkably downregulate the expression of p53. This probably indicates isoviolanthin has a good protective effect on human skin keratinocytes. Similarly, apigenin, a flavonoid from *Dendrobium officinale* significantly inhibits apoptosis and increases UVB-induced keratinocyte viability^[37,40]^ which led to much research conducted on the protection of apigenin on multiple skin problems.^[41]^ Nonetheless, the pharmaceutical and nutraceutical development of apigenin is greatly limited because it is quite insoluble in water. Fortunately, plants have evolved a mechanism for glycosylating flavonoids to increase their solubility.^[42]^ Coincidentally, isoviolanthin is the apigenin di-C-glycosides which contain arabinose and glucose residues at C-6 and C-8 (Fig. 1A) and can be dissolved in methanol and water.^[43]^ Although a study has found that DcaCGT catalyzes the C-glycosylation of apigenin,^[44]^ it is not clear which subtype C-glycosyltransferases (CGTs) catalyzes the conversion of apigenin to isoviolanthin.

Based on transcriptome analysis, this study found isoviolanthin treatment affected more than 2953 DEGs in injured keratinocytes, which were mainly attributed to 6 pathways, including DNA replication, Mismatch repair, RNA polymerase, Fanconi anemia pathway, cell cycle, and p53 signaling pathway. These pathways are involved in DNA damage and repair. Among them, the p53 pathway is a classical pathway, whose role in inducing cell apoptosis is widely accepted. P53 is considered to be the guardian of the genome, which can promote cell cycle capture, DNA damage repair, multiple cell death, and metabolic changes.^[31]^ Many studies have shown that the activation of p53 promotes apoptosis and autophagy of keratinocytes.^[45,46]^ The levels of p53 protein are very low in the unstressed state because it is degraded by the proteasome targeted by the E3 ubiquitin ligase MDM2. When cells are in the stressed or damaged state, the p53 protein rises significantly because activation of multiple signaling pathways inhibits MDM2.^[32]^

Among the top 30 differentially up-regulated genes, we noticed that *SPRR1A* and *SPRR1B, SLURP2,* and *KRT16* were significantly up-regulated, which is consistent with the previous study.^[33,34,47]^ Meanwhile, *KDM6B*, an important epigenetic regulator, is also remarkably up-regulated. *KDM6B* was involved in various cellular processes, including differentiation, proliferation, and apoptosis.^[48]^ It reported that *KDM6B* expression could be induced by mitochondrial stress inducers.^[49]^ *SPRRs* are antibacterial proteins produced in the epidermis, which protect the skin barrier by directly destroying bacterial cell membranes.^[35]^ In addition, *SPRRs* not only play an important role in keratinocyte homeostasis but also promote cell migration and wound healing in keratinocytes, and induce epithelial-mesenchymal transition in cholangiocytes. Furthermore, *SPRPs* play a vital role in free radical cleansing, directly protecting chromosome damage and the p53 signaling pathway.^[50]^ *SLURP2*, a psoriasis-related gene was found to be upregulated in the hyperproliferative skin cells.^[51]^ Researchers have found that treating keratinocytes with IL-22 increased *SLURP2* expression, which may be related to skin homeostasis or pathological formation of skin diseases.^[47]^ *KRT16* is a member of the keratins which are the major structural intermediate filament proteins in keratinocytes and are expressed in a specific pattern in different subtypes. Overexpressed *KRT16* could rescue the *KRT5*^-/-^ and *KRT14*^-/-^ mice that would die soon after birth due to blister formation.^[52]^ Among the top 30 genes that were considerably down-regulated, we discovered that the matching proteins of *CHAC2, ESCO2*, and *IPO4* could form stable complexes with isoviolanthin and have strong binding ability. Particularly, CHAC2 is involved in the degradation of intracellular glutathione and can be induced under endoplasmic reticulum stress, while the depletion of glutathione is a hallmark of apoptosis and it has been proven to be linked to many diseases.^[53]^ Furthermore, ESCO2 is involved in the acetylation of cohesins which are infinitely instrumental in DNA repair and chromatin structure during the cell cycle.^[54]^ The capacity of cohesins to bind sister chromatids is dependent on the acetylation of its Smc3 subunit. In addition, IPO4 is a primary nuclear input receptor of the histone H3 and H4, as well as their cytoplasmic partners, which are required for DNA integrity.^[55]^ It has been discovered that blocking IPO4 can prevent DNA damage caused by DNA-PKcs in cervical cancer.^[56]^ Finally, *MCM10* performs a critical regulatory role in the DNA replication timing and is a scaffold protein that stimulates DNA replication and protects it from replication stress.^[57]^ It reported that *MCM10* expression was higher in the model group than in the blank control group in the TNF-α induced psoriatic HaCaT cell model.^[36]^ On the whole, these genes abovementioned are involved in DNA replication, mismatch repair, cell cycle, and p53 signaling pathway.

Currently, there is little pharmacological and medical research on the isoviolanthin. In this study, the protective effect of isoviolanthin on injured skin keratinocytes through the p53 pathway was first found. The deeper mechanism may be that p53 pathways mediated jointly by targeting KDM6B, CHAC2, ESCO2, and IPO4 exert a skin protective effect, which is worthy to be further investigation.

## Author contribution

**Conceptualization:** Yanwei Xiang.

**Data curation:** Wei Zhu, Qingyi He.

**Formal analysis:** Haitang Zhang.

**Methodology:** Yunxiao Qiao.

**Resources:** Yanwei Xiang.

**Validation:** Lu Sun.

**Writing – original draft:** Jie Wang, Yin Hao.

**Writing – review & editing:** Yanwei Xiang.

## Supplementary Material
