# Supplementary material for: Research on the resistance of isoviolanthin to hydrogen peroxide-triggered injury of skin keratinocytes based on Transcriptome sequencing and molecular docking
Source: Medicine (Baltimore). 2023 Nov 24;102(47):e36119. doi: 10.1097/MD.0000000000036119 (PMC10681389; doi:10.1097/MD.0000000000036119)
Supplement: Supplementary file 1 [file medi-102-e36119-s001.docx]

**Supplementary Material for**

**Research on the resistance of** **natural flavonoid, isoviolanthin from *Dendrobium officinale，to* hydrogen peroxide-triggered injury of skin keratinocytes based on Transcriptome sequencing**


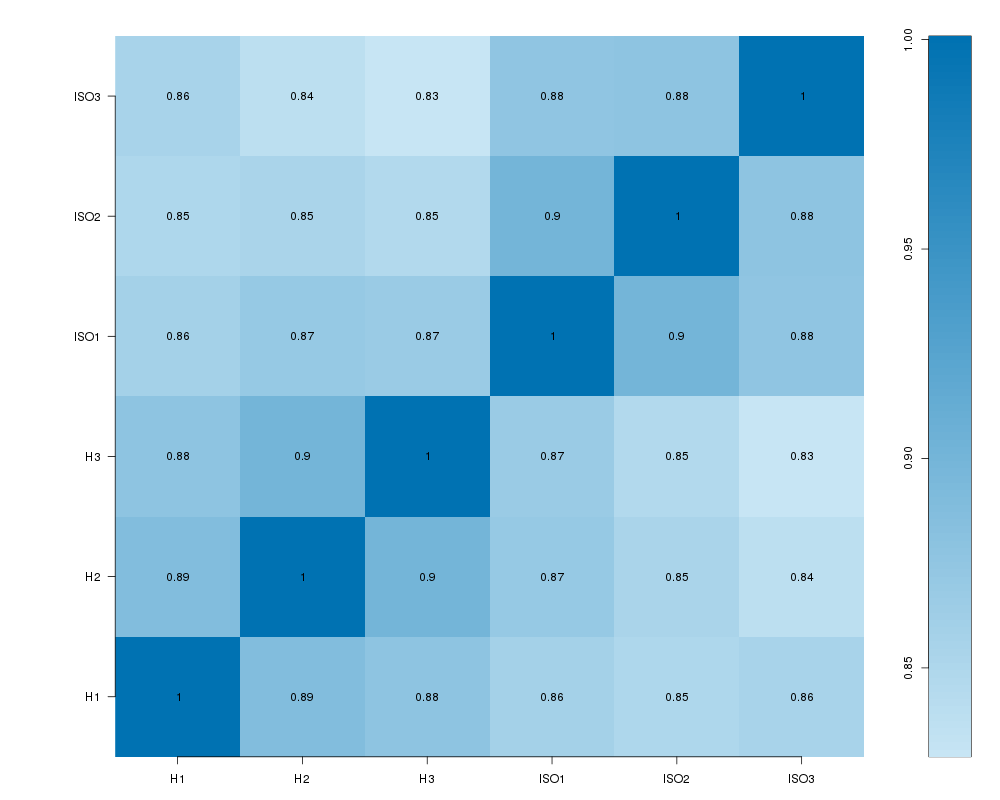


Figure S1.Correlations between sequencing samples. The correlation is stronger if the value in the grid is closer to 1
