# Supplementary material for: Research on the resistance of isoviolanthin to hydrogen peroxide-triggered injury of skin keratinocytes based on Transcriptome sequencing and molecular docking
Source: Medicine (Baltimore). 2023 Nov 24;102(47):e36119. doi: 10.1097/MD.0000000000036119 (PMC10681389; doi:10.1097/MD.0000000000036119)
Supplement: Supplementary file 2 [file medi-102-e36119-s002.docx]

**Supplementary Material for**

**Research on the resistance of** **natural flavonoid, isoviolanthin from *Dendrobium officinale，to* hydrogen peroxide-triggered injury of skin keratinocytes based on Transcriptome sequencing**


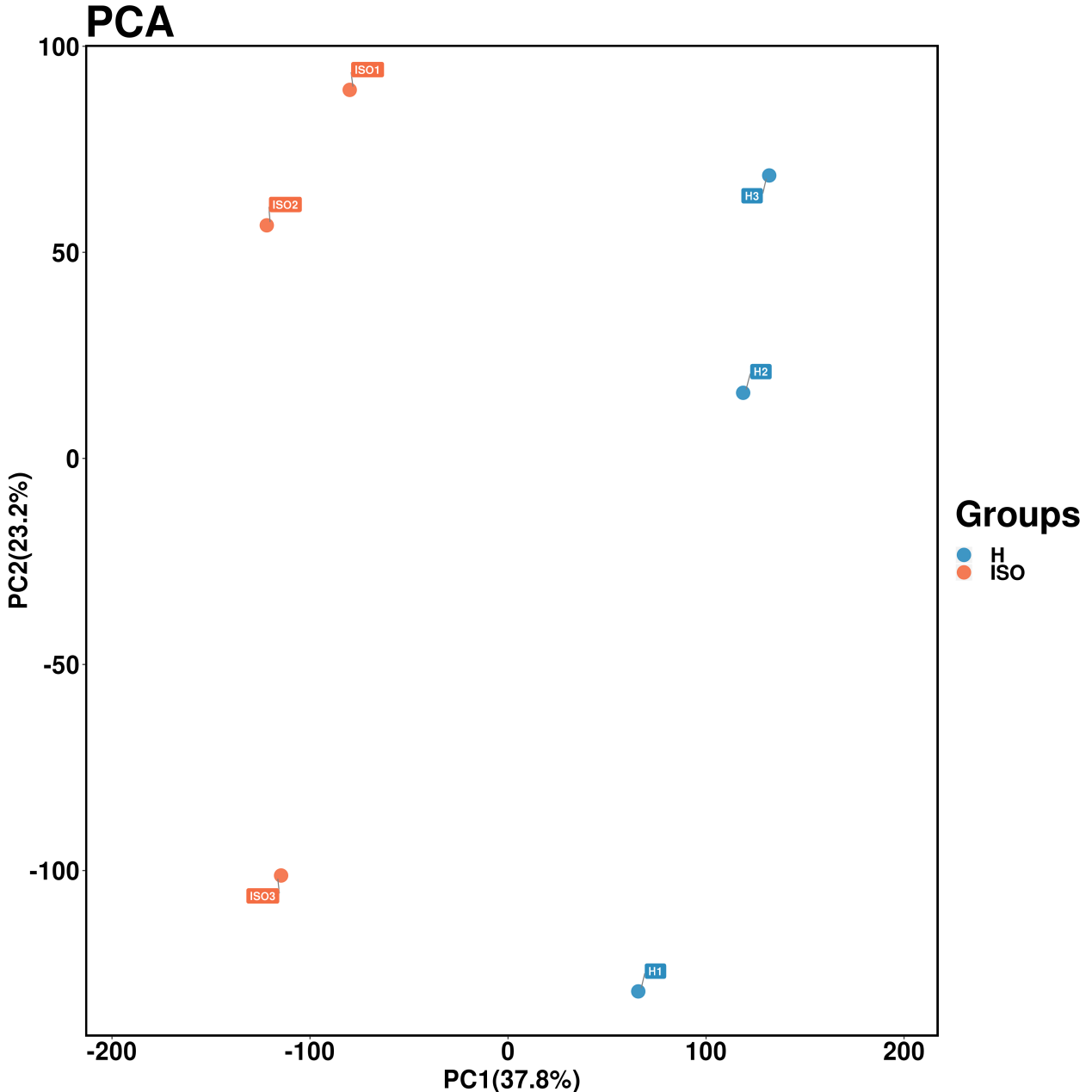


Figure S2.Principal component analysis diagram. Orange points represent “ISO group” and blue points represent “H group”.
